# Erythroderma Following Pfizer-BioNTech COVID-19 Vaccination in a Patient With Atopic Dermatitis: A Case Report

**DOI:** 10.7759/cureus.68936

**Published:** 2024-09-08

**Authors:** Marina Handal, Kawaiola Cael Aoki, Simona Bartos

**Affiliations:** 1 Medicine, Dr. Kiran C. Patel College of Osteopathic Medicine, Nova Southeastern University, Fort Lauderdale, USA; 2 Dermatology, Imperial Dermatology, Hollywood, USA

**Keywords:** covid-19 vaccine, exfoliative dermatitis, mrna-based vaccine, mrna vaccine, pfizer-biontech vaccine, vaccine adverse events, vaccine safety

## Abstract

Cutaneous adverse reactions to mRNA COVID-19 vaccines in patients with preexisting dermatologic disease include bullous eruptions, pityriasis rubra pilaris, dermatomyositis, and granuloma annulare. Erythroderma is a rare but severe adverse reaction not previously seen. This case report describes the development of erythroderma in a 73-year-old male with a history of atopic dermatitis, with widespread erythema and scaling covering 95% of his body surface area, which developed sequentially after receiving each dose of the Pfizer-BioNTech (BNT162b2) COVID-19 vaccination. The patient's condition improved significantly with appropriate dermatologic treatment, including systemic and topical corticosteroids and dupilumab. Thus, it is imperative to recognize erythroderma as a potential side effect of the Pfizer-BioNTech COVID-19 vaccine, particularly in patients with preexisting dermatologic conditions. Early diagnosis and treatment are vital for managing this potentially life-threatening reaction and preventing severe complications.

## Introduction

Among the mRNA COVID-19 vaccination therapies, the Pfizer-BioNTech (BNT162b2) COVID-19 vaccine is most associated with cutaneous manifestations, including local injection site inflammation, urticaria, and morbilliform rash eruption [1,2]. However, such cutaneous eruptions following vaccination against SARS-CoV-2 are commonly seen in patients with preexisting dermatologic disease. These include the eruption of bullous disorders and various dermatoses, including pityriasis rubra pilaris, dermatomyositis, and granuloma annulare [1].

Erythroderma, or exfoliative dermatitis, following Pfizer vaccination, has yet to be reported. Erythroderma is an inflammatory disorder that results in generalized erythema and scaling of more than 90% of the body surface area (BSA) [3]. Patients with erythroderma may also develop onycholysis, scalp scaling, and diffuse keratoderma along the palms and soles. The incidence of erythroderma is 1/100,000 adults and occurs more commonly in males at an average age of 61 years [4,5]. Common causes of generalized erythroderma include contact dermatitis, connective tissue disorders, psoriasis, and pityriasis rubra pilaris. Atopic dermatitis predisposes patients to erythroderma development, although infrequently [4]. This case reports erythroderma, a novel and uncommon adverse reaction to the Pfizer vaccine, in a patient with a history of atopic dermatitis.

## Case presentation

**Figure 1:**
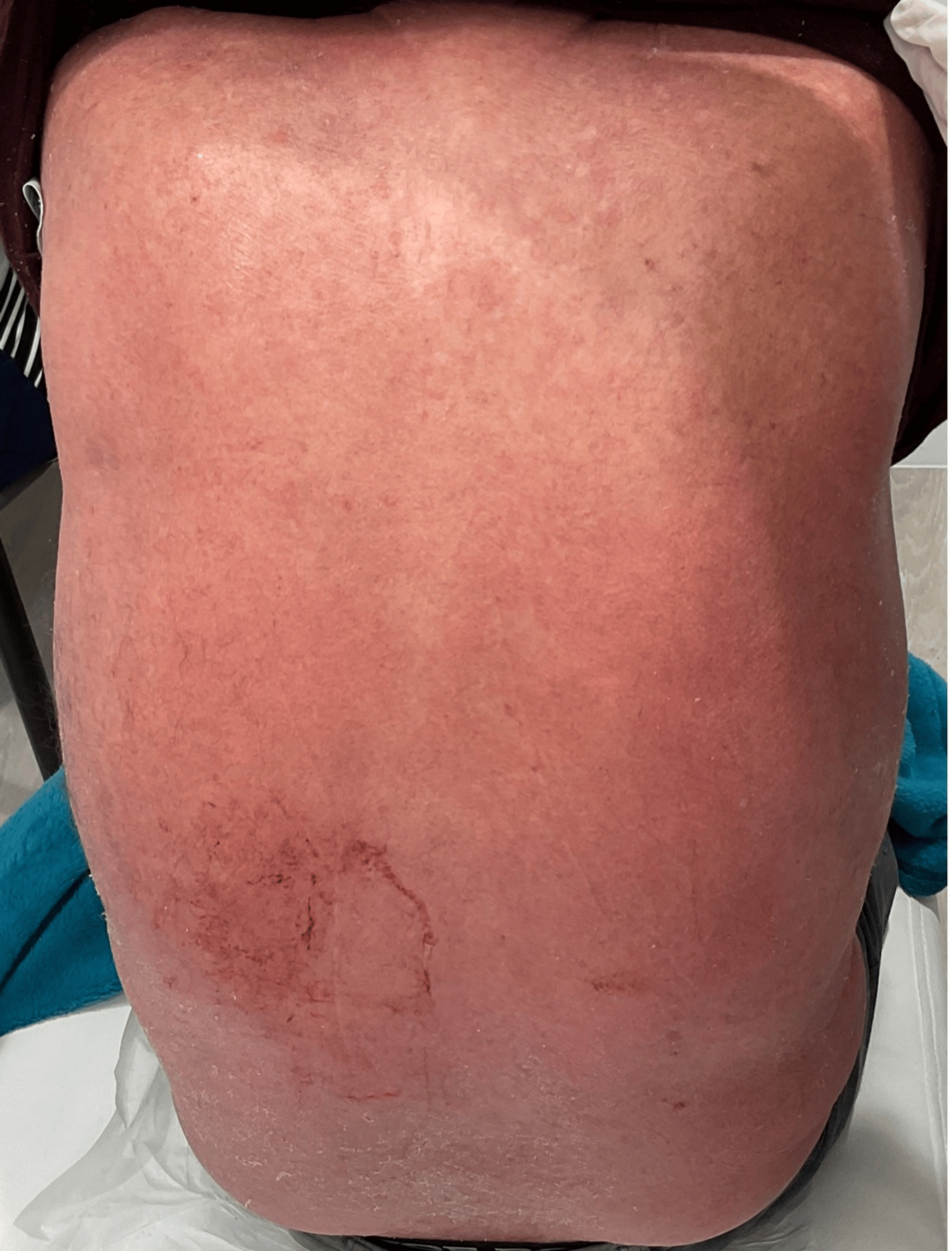
Widespread erythematous plaques on the back

**Figure 2:**
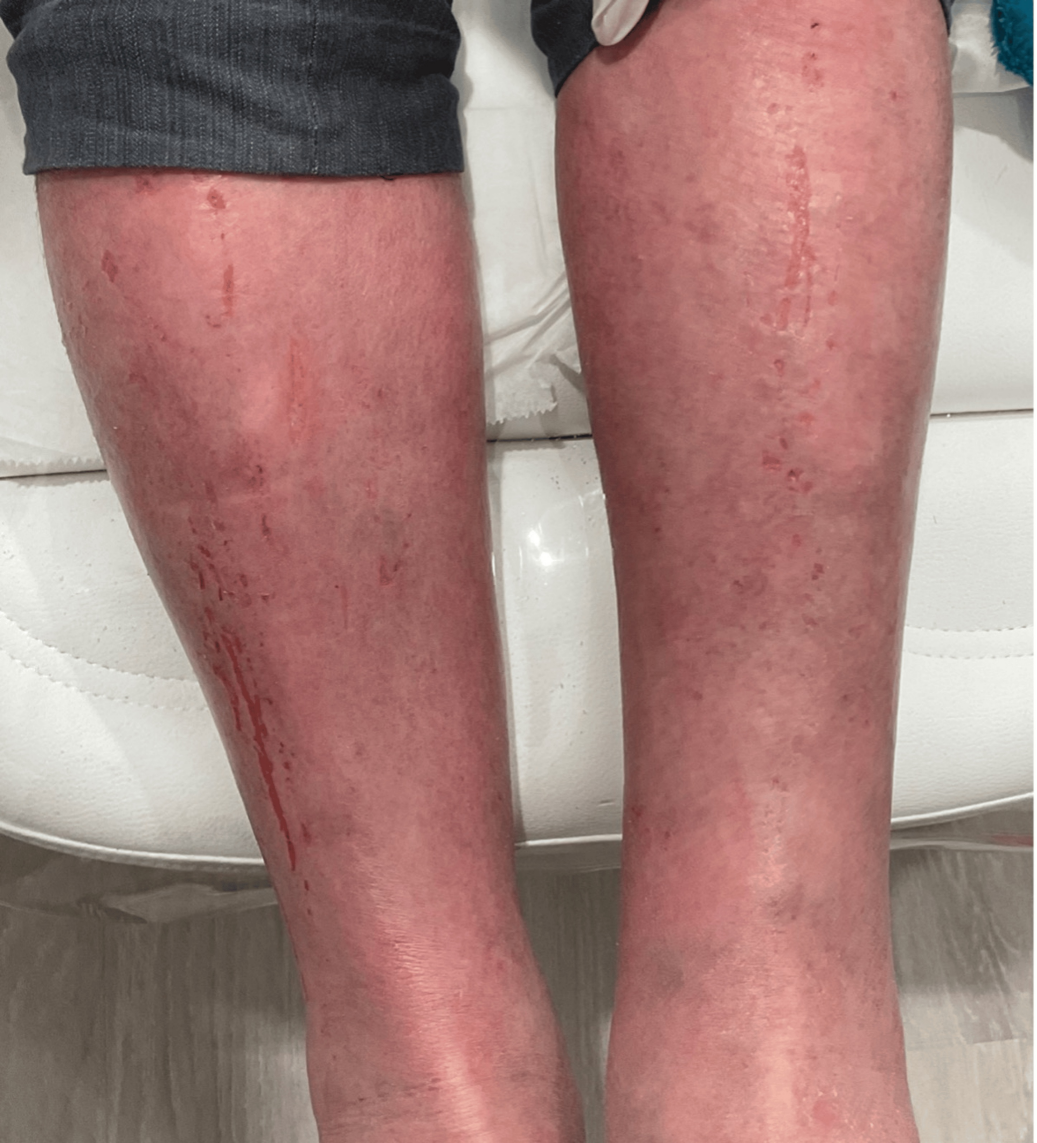
Diffuse erythema, excoriations, and bleeding on the legs

**Figure 3:**
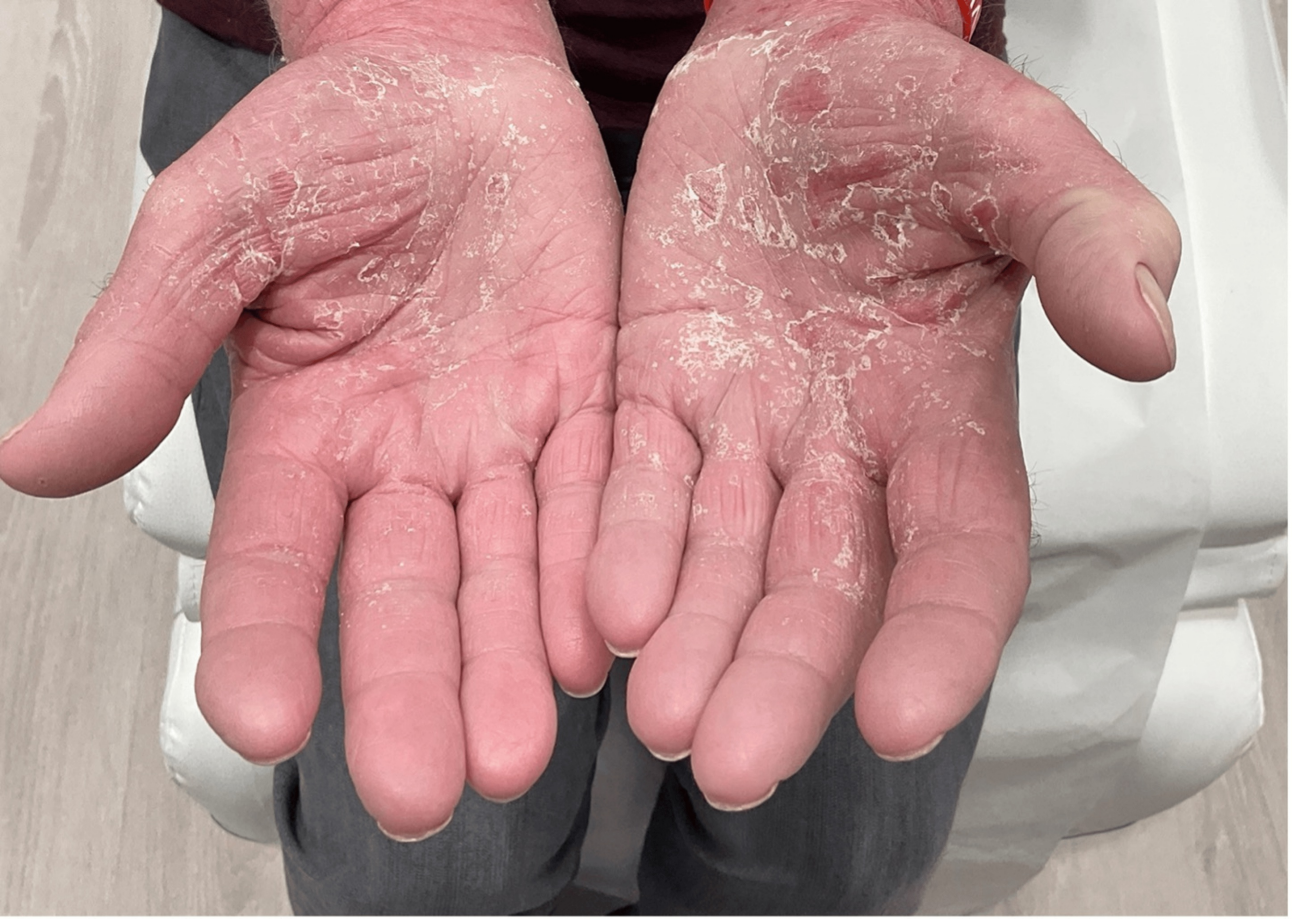
Erythematous and scaling lesions on the hands

A 73-year-old male with a history of atopic dermatitis presented to the clinic with bleeding skin, dryness, multiple ecchymoses, and itching for several days. He admitted to use of aspirin 81 mg for cardiovascular secondary prevention but denied other inciting factors, including changes to medications or using new topical products. The rash first appeared one month after receiving his first dose of the Pfizer vaccine. Following his second vaccination four weeks later, the rash became more diffuse, ultimately triggering a systemic rash with diffuse erythema across his body. Initial examination revealed erythematous, eczematous plaques with widespread desquamation and scaling along his neck, chest, abdomen, back, extremities, and gluteal regions, covering approximately 90% of his BSA (Figure 1, Figure 2, and Figure 3). There was no palpable lymphadenopathy. The diagnosis of erythroderma was given.

Dupilumab (300 mg/2 mL) injection was administered along with 1 mL of triamcinolone (40 mg/mL). The patient's body was covered with triamcinolone acetonide 0.1% ointment, and the affected areas were covered with plastic wrap. He was also given prescriptions for hydroxyzine 50 mg three times daily for seven days, prednisone 40 mg orally for 14 days, and triamcinolone 0.1% ointment twice daily for 30 days. Erythroderma secondary to atopic dermatitis requires meticulous management due to the extensive skin involvement and the risk of significant complications. In this case, aggressive systemic therapy was employed as a priority to prevent the need for immediate hospitalization. The patient was closely monitored with 24-hour follow-up to ensure a swift response to treatment and to address any potential adverse effects. To further support the patient's condition, plastic wrap was applied to the affected areas to minimize fluid loss and stabilize body temperature, both of which can be severely impacted by extensive skin involvement. Additionally, the patient was regularly assessed for signs of dehydration and electrolyte imbalance.

**Figure 4:**
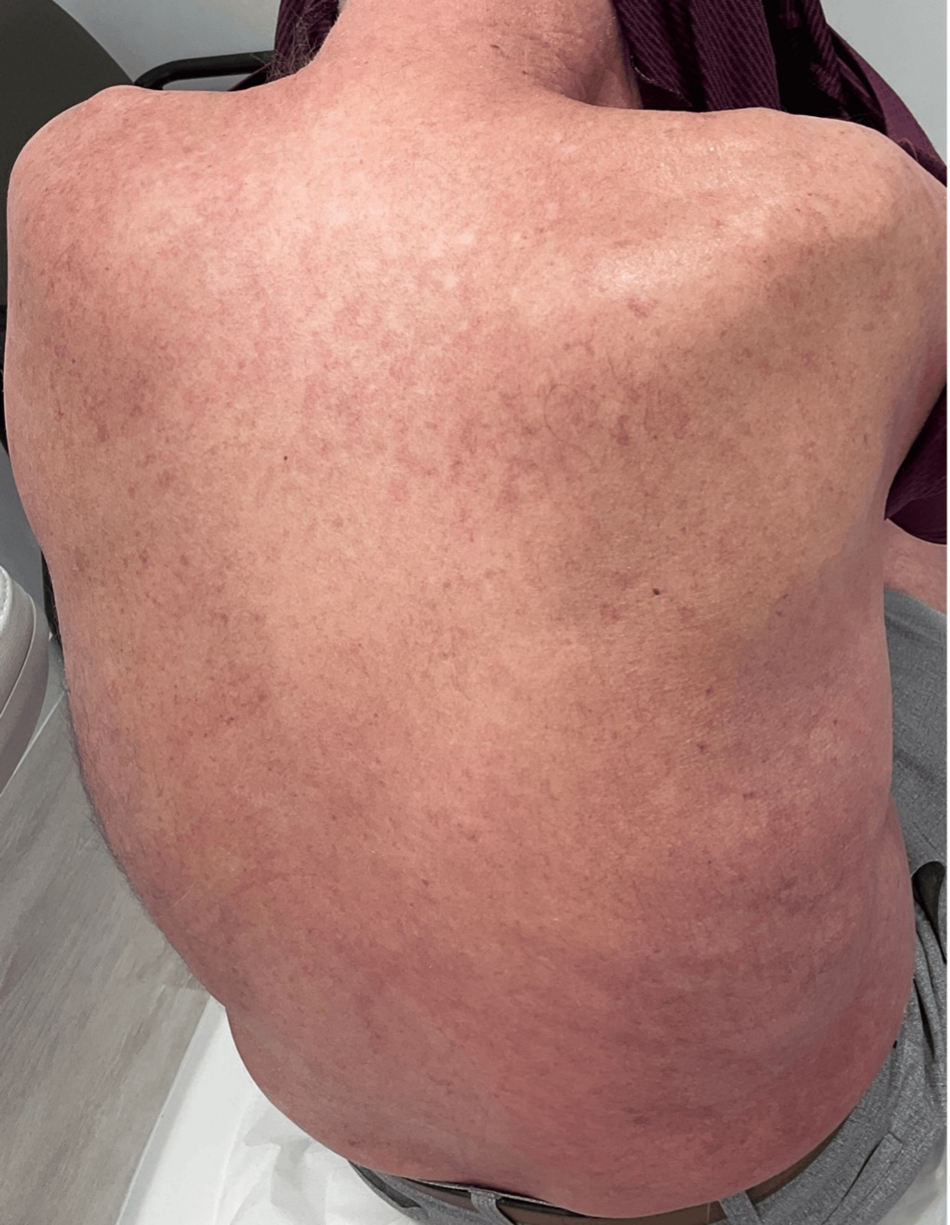
Significant improvement in erythema on the back after treatment

**Figure 5:**
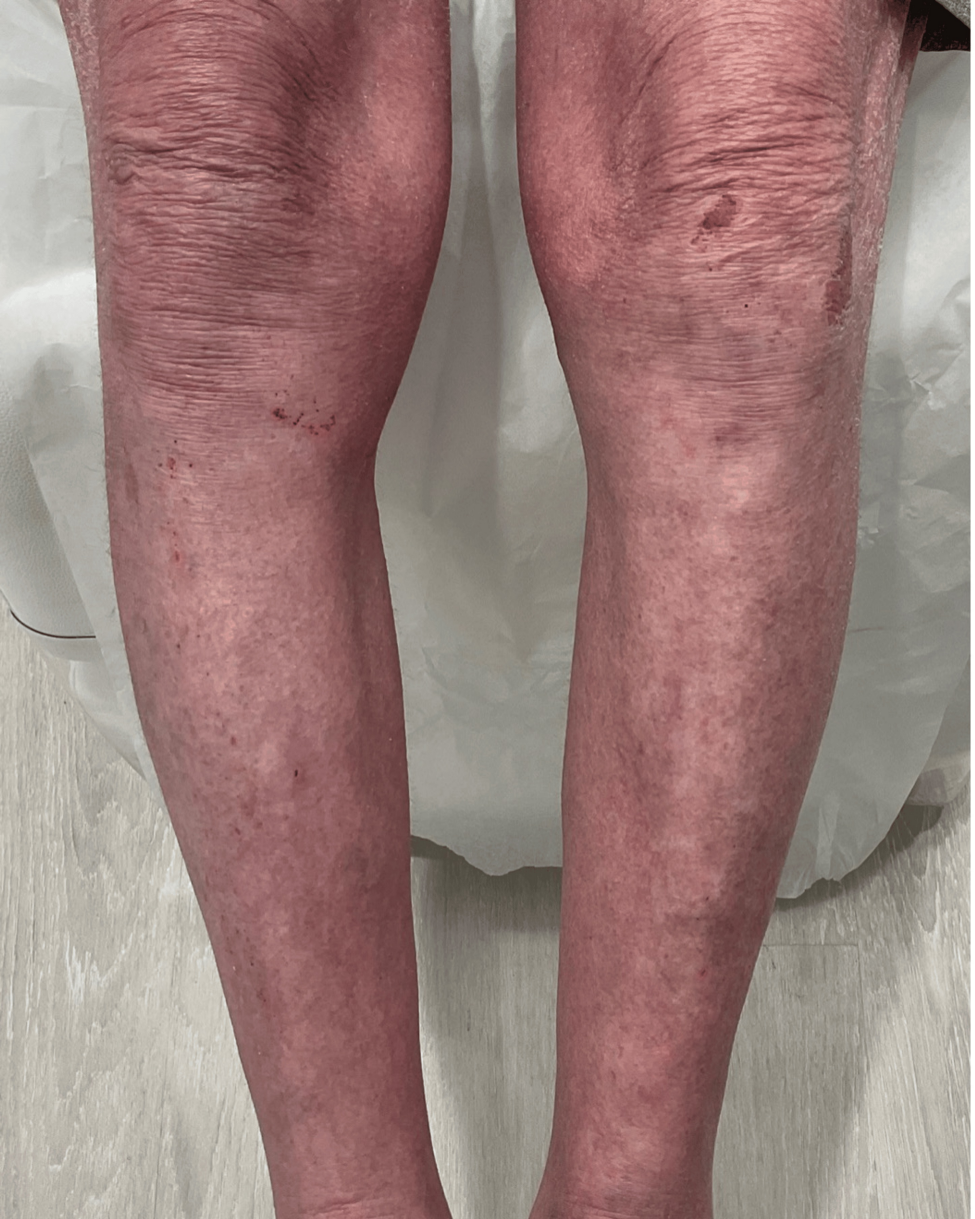
Resolution of erythematous lesions on the legs at four weeks

**Figure 6:**
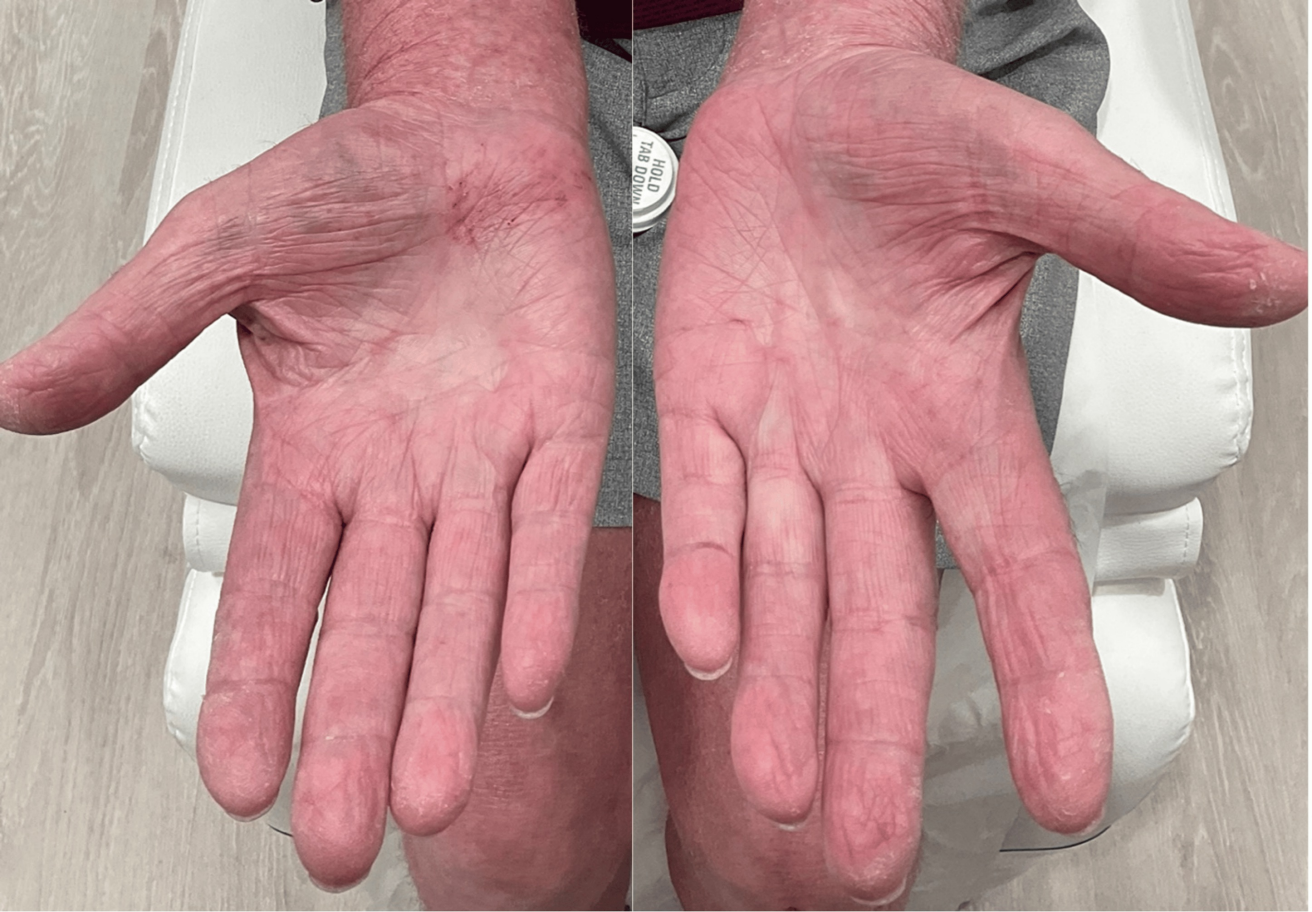
Resolved erythema and scaling on the hands at four weeks

At the following appointment two weeks later, the patient received a second injection of subcutaneous dupilumab (300 mg/2 mL). One week later, the rash had almost entirely resolved except for the lower abdomen. The patient was advised to continue a slower taper of prednisone for the next four weeks, and his symptoms were entirely resolved (Figure 4, Figure 5, and Figure 6).

## Discussion

This case presents the first systemic erythrodermic reaction following the Pfizer-BioNTech (BNT162b2) COVID-19 vaccination in a patient with a history of atopic dermatitis. The patient's clinical presentation, rash onset post-vaccination, history of atopic dermatitis, and the absence of changes to medication and topical regimens led to the conclusion that the Pfizer vaccine triggered a diffuse erythrodermic eruption. The combination of symptoms and sequential disease worsening with each dose supports the diagnosis of erythroderma due to mRNA vaccination.

Diagnosing erythroderma is challenging, as it is usually a manifestation of an underlying pathology or primary disorder. A comprehensive medical history and physical examination are critical in identifying possible etiologies and precipitating factors. Patients must be asked about previous rashes, allergy history, preexisting medical conditions, and medication use. More than 50% of patients with erythroderma have a medical history significant for localized skin lesions or underlying disease [6]. Our patient's sole medical history of atopic dermatitis, recent COVID-19 immunization, unremarkable medication use, and lack of systemic symptoms further corroborate a diagnosis of erythroderma secondary to Pfizer vaccination. However, a skin biopsy is warranted in cases where the etiology remains uncertain to enhance diagnostic accuracy [4].

Exacerbation of preexisting dermatoses is the most common trigger of erythroderma, including atopic dermatitis, pemphigus foliaceus, pityriasis rubra pilaris, and psoriasis, with atopic dermatitis being the most frequent association. Cutaneous lymphomas such as mycosis fungoides and Sézary syndrome may also be implicated, with histopathology demonstrating extensive monoclonal T-cell proliferation [4]. Patients with lymphoproliferative diseases often display systemic signs of infection, including lymphadenopathy, hepatomegaly, and splenomegaly [6].

Adverse drug reactions are the second most common cause of erythroderma, comprising about 20% of cases, and are often associated with allopurinol, phenytoin, and sulfonamides. These drug-induced reactions are characterized by morbilliform exanthems and erythema followed by scaling of the skin and papule eruption [6]. Psoriasis exacerbations following COVID-19 vaccination have also been reported, including plaque, palmoplantar, and guttate subtypes [7]. Generalized erythrodermic psoriasis (GEP) following mRNA vaccination is rare, with only nine cases reported. One case describes GEP in a patient with a 20-year history of psoriasis following the first dose of the Pfizer-BioNTech vaccine. This patient presented with total-body erythema and desquamating psoriatic plaques along the extremities [8,9]. Vaccine-induced erythrodermic pityriasis rubra pilaris has been reported, albeit limitedly [10-14].

The management of erythroderma depends on the underlying cause, and specific treatment is initiated accordingly. In cases of drug-induced erythroderma, low- to mid-potency topical corticosteroids and withdrawal of the offending drug are usually sufficient. Oral antihistamines and antibiotics may be used as adjunct therapy. Management of systemic symptoms, including nutritional support and fluid and electrolyte replacement, must also be considered [4].

## Conclusions

This case report underscores the need for dermatologists to recognize erythroderma as a potential adverse effect of the Pfizer-BioNTech (BNT162b2) COVID-19 vaccine. Because erythroderma poses a significant risk for dangerous and systemic complications, such as thermoregulatory disturbances and increased risk of cardiac failure, it is critical to elucidate the expansive dermatologic side effect profile of vaccination therapies in general. While erythroderma is a rare reaction to the Pfizer-BioNTech (BNT162b2) COVID-19 vaccine, it remains crucial for physicians, including primary care providers, to counsel patients on all potential risks and benefits. All adverse effects should be communicated to ensure patients are fully informed.
